# Coronavirus Disease 2019 and the Thyroid - Progress and Perspectives

**DOI:** 10.3389/fendo.2021.708333

**Published:** 2021-06-24

**Authors:** Hidefumi Inaba, Toru Aizawa

**Affiliations:** ^1^ The First Department of Medicine, Wakayama Medical University, Wakayama, Japan; ^2^ Department of Diabetes, Endocrinology, and Metabolism, Japanese Red Cross Wakayama Medical Center, Wakayama, Japan; ^3^ Diabetes Center, Aizawa Hospital, Matsumoto, Japan

**Keywords:** coronavirus, SARS-CoV, SARS-CoV-2, thyroid gland, autoimmunity, hyperinflammatory syndrome, cytokine storm

## Abstract

SARS-CoV-2 infection (COVID-19) is currently a tremendous global health problem. COVID-19 causes considerable damage to a wide range of vital organs most prominently the respiratory system. Recently, clinical evidence for thyroidal insults during and after COVID-19 has been accumulated. As of today, almost all non-neoplastic thyroid diseases, i.e., Graves’ disease, Hashimoto’s thyroiditis, subacute, painless and postpartum thyroiditis, have been reported as a complication of COVID-19, and causality by the virus has been strongly implicated in all of them. Similar thyroid problems have been reported in the past with the SARS-CoV outbreak in 2002. In this review, we briefly look back at the reported evidence of alteration in thyroid functionality and thyroid diseases associated with SARS-CoV and then proceed to examine the issue with COVID-19 in detail, which is then followed by an in-depth discussion regarding a pathogenetic link between Coronavirus infection and thyroid disease.

## Introduction

Coronavirus disease 2019 (COVID-19): caused by SARS-CoV-2 infection had broken out in China in December 2019 and then rapidly spread all over the world creating a global pandemic (1). Patients with COVID-19 suffer from high mortality due not only to respiratory failure but also to other complications such as cardiovascular collapse and disseminated intravascular coagulation (2, 3). In addition, there have been co-morbidities including autoimmune diseases associated with COVID-19 such as Guillain–Barre’s syndrome (4), autoimmune hemolytic anemia (5), and autoimmune thrombocytopenic purpura (6). Recently, evidence has been accumulated for changes in thyroid function and thyroid diseases associated with COVID-19 (7–29). Review articles on this topic have also been rapidly published (30–32). However, as far as we are aware, none of these have provided a thorough and comprehensive clinical picture and systematic etiological view on these thyroid complications. In this review, therefore, we performed a literature survey and displayed a balanced overview on the mechanisms involved in the thyroidal complications associated with COVID-19. The literature search was conducted up until March 30th, 2021, by using the keywords [COVID-19], [SARS-CoV-2], and [coronavirus] in combination with [thyroid], [thyroiditis], [thyrotoxicosis], [hyperthyroidism], and [hypothyroidism] through MEDLINE and PubMed. Only articles in English were cited. The review will begin with confirmation of what we have learned in the 2002 outbreak of severe acute respiratory syndrome (SARS) coronavirus (SARS-CoV) which is a platform for understanding the thyroidal problems associated with COVID-19.

In this communication, the terms “to provoke and provocation” were used to let the autoimmune abnormalities occur in the subjects without history of autoimmune thyroid disorders (AITD). On the other hand, “to activate and activation” were used to mean to arouse clinically silent, otherwise dormant, autoimmune abnormalities in those with history of AITD.

## Alteration in Thyroid Functionality During SARS-CoV Infection

Coronaviruses (CoVs), a family of single-stranded RNA viruses, typically cause signs and symptoms resembling common cold. The CoVs are subdivided into four genera2 such as Alphacoronavirus, Betacoronavirus (βCoV), Gammacoronavirus, and Deltacoronavirus (33).

Since both SARS-CoV and SARS-CoV2 belong to the same β-Coronavirus group, sharing the key clinical manifestations in common (33).

Alteration of the thyroid functionality has been documented in patients with SARS during the 2002 outbreak. We have summarized the similarities and dissimilarities between SARS and COVID-19 in **Supplementary Table 1**. Transient subclinical thyrotoxicosis, central hypothyroidism, and primary hypothyroidism were previously reported in patients with SARS (34). Wei et al. reported that thyroid-stimulating hormone (TSH) and adrenocorticotropin (ACTH) staining of thyrotrophs and corticotrophs, respectively, was significantly attenuated in the pituitary gland of patients with SARS upon autopsy (35). Apoptosis of the thyroid follicular cells was seen in SARS (36), but the information on the thyroid function of the patients was not provided (36). SARS-CoV virus has not been found in the thyroid gland (37) and, therefore, primary hypothyroidism in this patient may or may not have been a consequence of the direct viral attack on the thyroid follicular cells. In addition, the non-thyroidal-illness syndrome (NTIS) due to severe acute respiratory distress in SARS-CoV infection has been implicated (38) **(** **Supplementary Table 1** **).**

## Thyroidal Insults Observed in COVID-19 and Their Management

**Figure 1:**
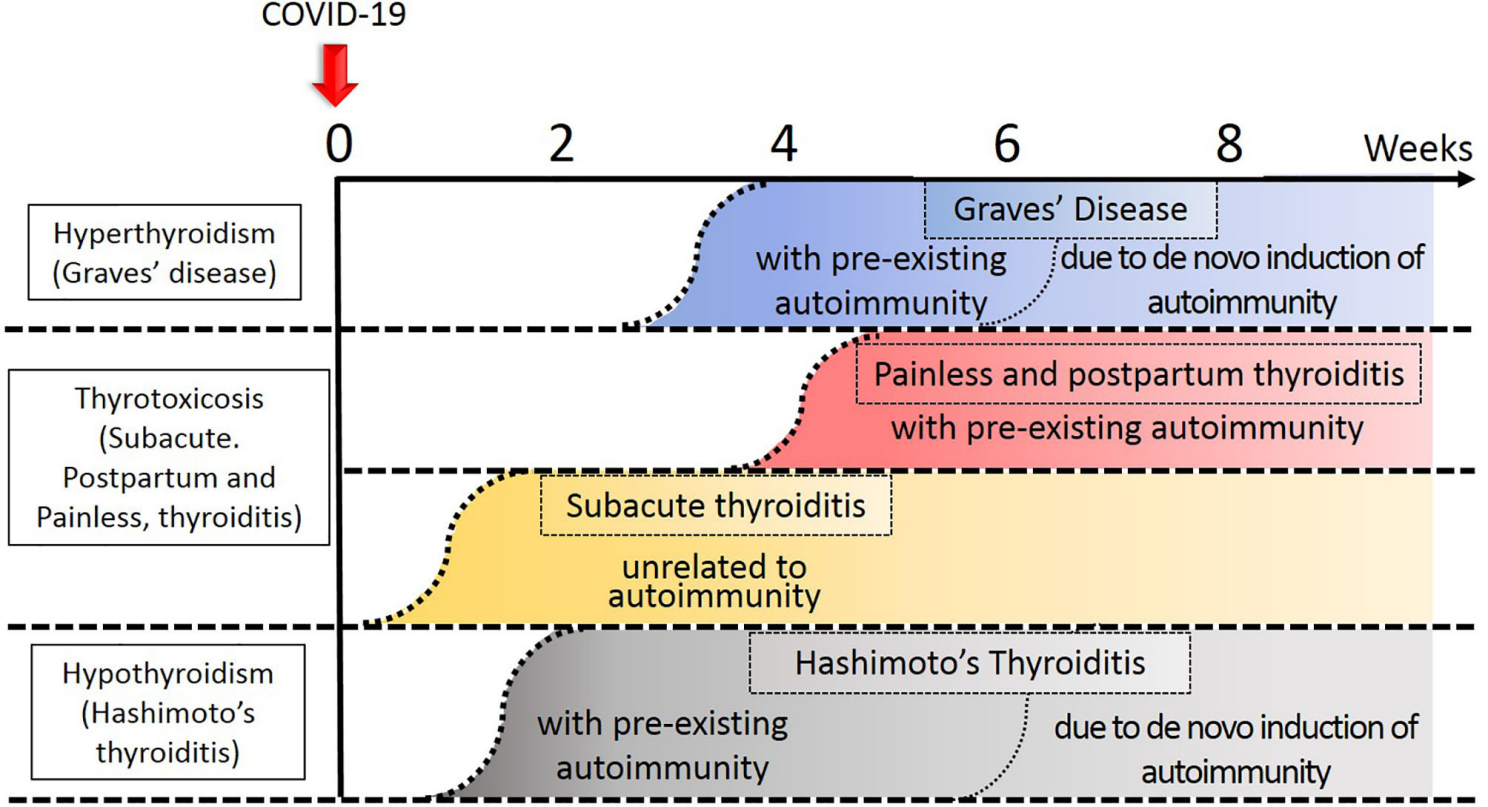
Temporal profile of development of thyroid diseases in relation to COVID-19. Approximate timing of onset of each disease was indicated by the curvilinear broken lines. Thyroid disease process related to autoimmunity tend to occur earlier in the subjects with preexisting autoimmunity to the thyroid gland. The darker the color, the degree of thyroidal insults stronger.

We divide the consequences of COVID-19 into major and minor influences on the thyroid gland and its function (**Figure 1**).

### Major Sequelae

#### Destructive Thyroiditis

##### Subacute Thyroiditis (SAT)

This is an inflammatory disorder, usually with a painful goiter, fever, palpitation, and fatigue (39). Elevated serum levels of C-reactive protein (CRP) and erythrocytes sedimentation rate (ESR), together with focal hypoechogenic areas in the thyroid gland are characteristic laboratory findings of SAT (39). Damage to the thyroid per se and suppression of TSH by thyrotoxicosis together cause extremely low thyroidal uptake of radioiodine (39, 40). Nonetheless, thyrotoxicosis in SAT commonly subsides within 3 months (40). Viral infection such as human foamy virus (HFV), mumps, coxsackie, adenovirus, Epstein-Barr viruses (EBV), measles, chickenpox, cytomegalovirus (CMV), influenza, and rubella have all been implicated as a cause of SAT (40, 41). An association of HLA and SAT was also reported (40), and will be discussed in section Etiologic Background for Thyroidal Insults in COVID-19.

To date, a total of 13 cases of SAT with COVID-19 have been reported (**Supplementary Tables 2** and **3**) (7–16). Most reports (7/13, 54%) were from Italy (**Supplementary Table 3**). Patients were distributed across all generations (18-69 years old) with overwhelming female predominance (11/13, 85%). The onset of SAT ranged from ‘7 weeks before’ to ‘7 weeks after’ the diagnosis of COVID-19. Fever was a common symptom in the patients with COVID-19 irrespective of the presence or absence of SAT so that it cannot be taken as a sign of SAT in this situation. Accordingly, thyroidal pain and tenderness are diagnostic clues for SAT with the combination of thyrotoxic signs and symptoms such as palpitations, finger tremor, hyperhidrosis, and soft stool. The patients usually show increased serum levels of FT3 and FT4 concurrent with a decreased level of TSH. Izumi et al. previously reported that the mean (SD) value for the serum FT3 to FT4 ratio was 0.399 (0.089) in patients with GD, 0.335 (0.057) in those with SAT, and 0.304 (0.072) in painless thyroiditis (PT), respectively, so that the lower serum FT3/FT4 in the presence of thyrotoxicosis can be a marker for thyroiditis, not Graves’ hyperthyroidism (42). The mean value for FT3/FT4 in patients with SAT associated with COVID-19 ranged from 0.22-0.32 (**Cases 1, 2, 4, 5, 6, 7, 8, 10**), suggesting that “the Izumi hypothesis” was applicable in these patients (**Supplementary Table 3**). The serum levels of thyroglobulin (Tg) were increased in 4 patients (**Cases 1, 2, 6, 8**) and CRP levels were increased in all of the patients tested (**Supplementary Table 3**). A hypoechogenic area in the thyroid, either focal or diffuse, was found in the majority of them and echogenic evidence for increased vascularization was absent in all of the patients tested (except for case 12) (**Supplementary Table 3**). Thyroid scintigrams showed decreased uptake in all of the cases tested (**Cases 2, 6, 8, 12**) (**Supplementary Table 3**). Thyroid autoantibodies (TSH receptor autoantibody (TRAb), TgAb, and TPOAb) were seldom positive.

Muller et al. reported a high frequency of atypical SAT in their patients with COVID-19 (29) (**Supplementary Tables 2** and **3**). They reported that 13 out of *consecutive* 85 (15%) COVID-19 patients admitted to their high-intensive care units (HICU20) had overt thyrotoxicosis: the corresponding value was 1% in the HICU patients without COVID-19 (HICU19) and 2% among patients with COVID-19 in the low-intensive care units (LICU20). As an entire group, the mean (SD) FT4 level in the patients in HICU20 and LICU20 was, 18.7 (5.4) pmol/l and 13.5 (4.6) pmol/l, respectively: it was significantly higher in the former (*P* = 0.016). The authors named these patients ‘atypical’ because thyroidal pain and swelling were absent. Despite such ‘atypical’ physical findings, focal hypoechogenic areas, decreased ^99m^Tc uptake, and negativity of thyroid autoantibodies (except for one patient) were all present as in the typical subacute thyroiditis, which made them propose the new concept, ‘atypical SAT’ (29). Lania et al. reported that 58 out of consecutive 287 (20%) patients with COVID-19 hospitalized in non-ICU beds developed thyrotoxicosis possibly provoked by systemic inflammation or immune activation induced by COVID-19 (24) (**Supplementary Table 4**). Out of the 58 thyrotoxic patients, thyrotoxicosis was clinically overt in 31 (53%). On the other hand, 15 out of the 287 (5%) had hypothyroidism. Importantly, in the entire group, there was a strong, inverse correlation between the serum level of IL-6 and TSH (Spearman rho = -0.41, *P* < 0.001), indirectly suggesting at least a partial contribution of inflammatory destruction of the thyroid to the elevation of the serum thyroid hormone levels. Moreover, the patients with thyrotoxicosis (TSH < 0.34 mIU/l) had significantly higher levels of the mean serum IL-6 than those without thyrotoxicosis (*P* < 0.05) (most of them > 10 pg/ml with the reference range < 6.4 pg/ml), suggesting inflammation due to COVID-19 infection, as indexed by elevated IL-6, was a driving force for the thyroiditis. They concluded that COVID-19 may have provoked thyrotoxicosis in the 31 cases described above.

In summary, these reports (24, 29) indicated that thyrotoxicosis may occur in 10-20% of the patients with COVID-19. Increased levels of serum IL-6 were also reported by Bartalena et al. in cases of destructive thyroiditis (43). Moreover, IL-6 has been reported to be involved with various autoimmune and inflammatory diseases (44). Han et al. revealed that serum IL-6 levels can predict disease severity in patients with COVID-19 (45).

##### Management of SAT and Atypical SAT in COVID 19

SAT is treated with 16-40 mg/day prednisolone followed by tapering within 4-6 weeks (7–16). Atypical SAT is a self-limiting disorder, and therefore can be observed without specific pharmacological treatment (29).

#### Autoimmune Thyroid Disease (AITD)

##### Painless Thyroiditis (PT) and Painless Postpartum Thyroiditis (PPT)

These disorders may belong to destructive thyroiditis, and also may be subtypes of autoimmune thyroid disease (AITD) (40, 46, 47) although painless SAT may also occur if the inflammatory response is mild. In general, a majority of the patients with PT and PPT initially experience a mild thyrotoxic phase with elevated serum thyroid hormone levels and depressed TSH, which was followed by depressed thyroid function and then recovery to normal within several months, i.e., spontaneous resolution of the thyroid dysfunction. We hypothesize that autoimmune-associated thyroiditis with COVID-19 may preferentially be observed in the subjects who possess susceptibility to AITD, because the patients who experienced PT and PPT in association with COVID-19 often developed thyroid autoantibody positivity 1-1.5 months later (28). Such patients may share increased HLA genotypes with patients with AITD (See section *Etiologic Background for Thyroidal Insults in COVID-19*).

PPT patients who had both TPOAb and TgAb often have an increased percentage of activated T cells, such as HLA-DR+ and CD3+ cells, in the peripheral circulation (48). Thus, alteration in the T-cell population may be predisposed to or associated with the development of PPT. Along with this evidence, Elefsiniotis et al. encountered the development of PPT in 4 out of 16 chronic hepatitis C virus (HCV)-infected women, proposing “viral-triggered PPT” as a subtype of the thyroiditis (49). Altered T cell populations in patients with HCV infection have been considered as a reflection of Th1/Th2 imbalance (50).

##### Graves’ Disease (GD) and Hashimoto’s Thyroiditis (HT)

The AITD’s are a constellation of thyroid-specific autoimmune diseases, and Graves’ disease (GD) and Hashimoto’s thyroiditis (HT) are the two major disorders included in this entity (51, 52). GD is characterized by TSH receptor antibodies (TRAb) which stimulate the thyroid follicular cells leading to hyperthyroidism (51). HT is characterized by the positivity of the serum for thyroglobulin autoantibody (TgAb) and thyroperoxidase autoantibody (TPOAb) (52). Hypothyroidism in HT is due to T-cell mediated damage of thyrocytes and interstitial fibrosis. The serum thyroid autoantibodies such as TgAb and TPOAb are often also positive in patients with GD (53). Clinically, transitions of patient from GD to HT and vice versa are not uncommon (52, 54), and a positive family history for GD often overlaps with that for HT (55). Provocation or activation of AITD by COVID-19 toward the seemingly opposite direction, to GD or HT, can be understood from these perspectives (**Supplementary Figure 1**).

The association of viral and non-viral infection and AITD has often been suggested (56–58). For example, serological evidence of infection with human herpesvirus-6 (HHV-6) (56), and Toxoplasma gondii (57), HCV (58) was obtained from patients with AITD at, or around, the time of diagnosis of AITD. However, it remains to be determined whether the infection was causal for the development of the thyroid diseases or just innocent bystanders (41). The relationship between SARS and AITD also has not been described with certainty.

Two cases of GD with COVID-19 were reported by Mateu-Salat et al: one case was a relapse of hyperthyroidism in a 60-year-old woman in whom GD had been in the state of drug-free remission for longer than 30 years (25). The other was the development of GD in a 53-year-old woman without a known history of thyroid disease (25) (**Supplementary Table 2**). Cervical pain was absent and Graves’ ophthalmopathy was equivocal in both of these cases. Despite such ambiguity in signs and physical findings, the serum thyroid hormone levels were indeed elevated and TSH suppressed, thyroid iodine scintigram uptake increased and TRAb was positive, so that they were diagnosed as GD. The timing of the diagnosis of GD was 1 and 2 months after the onset of COVID-19 in the former and the latter, respectively. Harris et al. reported another case of GD (26) (**Supplementary Table 2**). A 21-year-old woman presented with tachycardia, palpitation, anxiety, and finger tremor 17 days after the diagnosis of COVID-19. Her mother had hypothyroidism. A diffuse, non-tender, moderate-sized goiter was present. Elevated thyroid hormone and suppressed TSH levels, and the positivity of TRAb indicated the diagnosis of GD. Graves’ ophthalmopathy or dermopathy was absent. The three patients with GD responded to thiamazole and β-blocker uneventfully.

A 45-year-old man with COVID-19 who presented with fatigue and muscle weakness was diagnosed as HT, based on the hypothyroidism with the positivity of TPOAb and successfully treated with 25 μg/day L-thyroxine (27) (**Supplementary Table 2**). As can be seen, in the patients with AITD (25–27), depending upon the different background thyroid autoimmunity, a variety of organ-specific autoimmune abnormalities may be provoked or activated upon SARS-CoV-2 infection.

Liu et al. reported that 25 out of a consecutive 191 (13%) patients with COVID-19 showed abnormal results in thyroid function tests (17) (**Supplementary Table 4**). Ten patients had isolated low TSH, suggestive of subclinical thyrotoxicosis: one of them was positive for TPOAb and TRAb, and two of them were positive for TRAb. Therefore, the three might have had subclinical GD. Apart from the ten patients, there was a patient with isolated high FT4 and another with high FT4 and low FT3, who were also positive for TRAb leaving the possibility of mild GD. Patients with abnormal thyroid function in this study contained additional three patients: the first one with isolated high FT4 with TgAb positivity, the second one with isolated TSH elevation with positive TPOAb and TgAb, and the third one with low TSH and FT3. The remaining ten patients with isolated low FT3 were compatible with non-thyroidal illness syndrome, and one patient was positive for TRAb and TPOAb (see section below on ‘*Non-Thyroidal Illness Syndrome (NTIS)*’ below). Overall, 14/191 (7%) had features of thyrotoxicosis, diagnosed by low TSH and/or raised FT4. The authors re-examined 10 of the 25 patients a median of 28 days after the initial thyroid function test and found normalization, permanent hypothyroidism, and various stages of thyroiditis in evolution, with no uniform recovery.

##### Management of AITD in COVID-19

PT and PPT are self-limiting disorders, and therefore can be observed without specific pharmacological treatment (29). Regarding the management of GD, treatment of thyrotoxicosis by thionamide drugs is usually safe, but should be performed with caution. This is because the signs and symptoms of COVID-19 are indistinguishable from those of antithyroid drug-induced agranulocytosis. On the other hand, hypothyroidism due to HT can be treated by a regular L-T4 supplement.

### Minor Sequelae

#### Non-Thyroidal Illness Syndrome (NTIS)

A systemic disease of any kind, if it is critical, causes the non-thyroidal-illness syndrome (NTIS), characterized by low T3 levels as a result of changes in type 1 deiodinase activity (38, 59). Patients in the ICU typically present with decreased levels of serum T3, normal or low T4, and normal or slightly decreased levels of TSH (38). Zou et al. reported that 41 out of 149 (28%) patients with COVID-19 fitted the diagnosis of NTIS (FT3 levels < 2.3 pg/ml) (19) (**Supplementary Table 4**). Similarly, Gao et al. showed that FT3 levels were significantly lower in patients with severe COVID-19 (66 out of 100: 66%) than in mild COVID-19 patients (34 out of 100: 34%) (20) (**Supplementary Table 4**).

In a consecutive evaluation of deceased (N = 113) and recovered (N = 161) COVID-19 patients, the serum levels of TSH and FT3 on admission were significantly lower in the former (21) (**Supplementary Table 4**). Taken together, a low level of FT3 is commonly associated with, or predictive, of an intractable form of COVID-19. Another group found that total T3 levels were inversely correlated with the severity of COVID-19 (22) (**Supplementary Table 4**). Nonetheless, the data should be interpreted carefully because a significant proportion of the patients (31/50: 62%) were taking glucocorticoid at the time of the hormone measurements (22).

Lui et al. (17) evaluated 191 COVID-19 patients cross-sectionally and found that 1) lower SARS-CoV-2 PCR cycle threshold levels and elevated CRP levels were associated with low levels of TSH, 2) elevated CRP was associated with low FT3, 3) elevated levels of ESR associated with lower FT3 to FT4 ratio, and 4) lowering of FT3 was associated with increasing COVID-19 severity. Somewhat differently, Khoo et al. reported that 289 out of 334 (87%) patients with COVID-19 were euthyroid (23) (**Supplementary Table 4**). They recognized that patients with COVID-19 had lower levels of TSH and FT4 compared to those who did not have COVID-19. They also reported that the serum TSH and FT4 concentration were both depressed upon admission for the treatment of COVID-19 compared to the pre-hospital baseline levels. They also reported that patients who were admitted to the ICU had lower TSH levels than those treated at the non-emergency ward. There was a significant inverse correlation between the serum cortisol and TSH levels and between CRP and TSH levels, and a positive correlation between CRP and FT4 levels. The findings suggested stress-induced suppression of TSH in COVID-19. At least partial recovery of TSH levels toward the baseline was observed at the follow-up at several months later (23). Elevated serum levels of cortisol in patients with COVID-19 were reported (60), and hypercortisolism has been reported to suppress TSH levels (23, 61). This change in TSH may be likely due to the changes in deiodinase activity in the central nervous system (38).

##### Management of NTIS in COVID-19

Since NTIS is due to the systemic dysfunction by COVID-19, the treatment for COVID-19 is essential to obtain normal thyroid functional test results (TSH, FT3, and FT4).

#### AITD Patients Response to COVID-19

Up to this point, the data regarding the effect of COVID-19 on the thyroid gland has been reviewed. Gerwen et al. investigated the problem the other way around. Namely, they identified 251 patients with hypothyroidism receiving thyroid hormone replacement among 3,703 patients with COVID-19 (251/3703, 7%), and concluded that hypothyroidism was not associated with an increased risk of hospitalization, mechanical ventilation, or death in patients with COVID-19 (18) (**Supplementary Table 4**). Unfortunately, the impact of COVID-19 on the pre-existing thyroid-related cellular and humoral immunity was not evaluated in this study. It is, however, possible on the basis of other reports (50, 62), that viral infection is likely to exaggerate the Th1/Th2 imbalance and reduce T regulatory cell (Treg) action (63) causing it to initiate a flare-up otherwise clinically dormant AITD explaining some of the cases described but there remains no evidence that it exacerbates already established disease. In fact, impairment of Treg has also been shown in AITD (64).

## Etiologic Background for Thyroidal Insults in COVID-19

### Major Sequelae

#### Hyperinflammatory Syndrome

**Figure 2:**
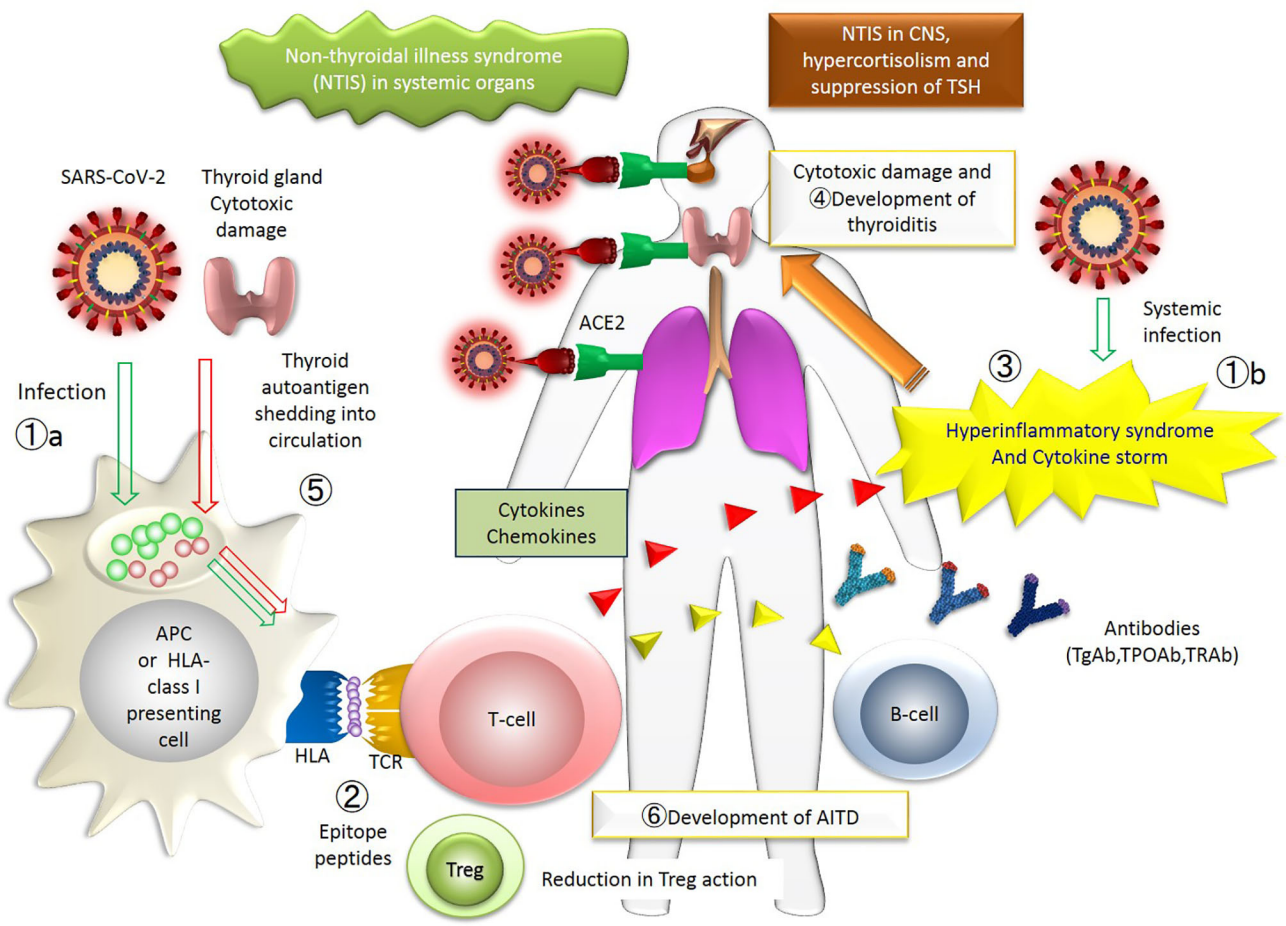
The figure represents the whole picture of the thyroid insults during COVID-19 in the immunological network. The events occurring in patients with thyroid diseases associated with COVID-19 are collectively shown. The numbers approximately correspond to the sequence of events. ①. SARS-CoV-2 infects systemic organs through acquired immunity (①a) and innate immunity (①b). ②. SARS-CoV-2 epitope peptide is presented on the surface of HLA, and T-cell recognizes the epitope peptide. ③. Hyperinflammatory syndrome and cytokine storm occur. ④. Thyroid gland is damaged by immune cells. ⑤. Thyroid autoantigen is shedding into circulation, which is also presented on the surface of HLA. ⑥. Finally, AITD develops as a consequence of new provocation of the disease or activation of previously existing dormant disease. APC, antigen-presenting cells; TCR, T-cell receptor; T-cells include cytotoxic T-cells and effector T-cells; AITD, autoimmune thyroid disease. A putative mechanism for the thyroid insults during COVID-19: an immune-centric view. The numbers approximately correspond to the sequence of events. ①, SARS-CoV-2 infects systemic organs through acquired immunity (①a) and innate immunity (①b). ②, SARS-CoV-2 epitope peptide is presented on the surface of the antigen-presenting (or HLA class I) cells, and T-cell recognizes the epitope peptide. ③, Hyperinflammatory syndrome and cytokine storm occur. ④, Thyroid gland could be a target of the antibodies or T-cells. ⑤,Thyroid autoantigen is shedding into the general circulation, which is also presented on the surface of the HLA-cells. ⑥, Finally, AITD develops as a consequence of the new provocation of the disease or activation of the pre-existing, yet dormant disease. APC, antigen-presenting cells; TCR, T-cell receptor; T-cells include cytotoxic T-cells and effector T-cells; Treg, regulatory T-cell; AITD, autoimmune thyroid disease.

Increased proinflammatory/Th1 cytokine production has been associated with respiratory failure due to lung damage from inflammation of the pulmonary tissue in SARS (65). Huang et al. found that COVID-19, especially in its severe form, is associated with a hyperinflammatory syndrome characterized by similar hypercytokinemia with multiorgan failure as seen in SARS (2) (**Figure 2**). They have also reported that, in COVID-19, proinflammatory/Th1 cytokine production is increased and Th2 cytokines increased as well, which was a different picture from SARS (2). A pathogenetic role of cytokines in the development of thyroiditis and flare-up of the thyroid autoimmunity has been implicated (66). We hypothesize that dormant AITDs such as GD and HT become clinically overt by Th2-mediated autoantibody production and Th1-mediated cellular immunity, respectively, in COVID-19 and exaggerated further by Treg dysfunction (**Supplementary Figure 1** and **Figure 2**).

As described above, Lania et al. reported a significant positive correlation between serum IL-6 and the degree of thyrotoxicosis in patients with COVID-19 (24). The finding suggested that elevation of IL-6 and/or cytotoxic effects of T-cells during the hyperinflammatory syndrome might be causal for thyroidal destruction of the thyroiditis in COVID-19 (2, 24, 28, 29).

They further postulated that the thyroiditis may not subside as long as the storm persists. Such patients may end up with permanent hypothyroidism or thyrotoxicosis years later (24). As a conclusion to this part, the hyperinflammatory syndrome in COVID-19 appears to provoke AITD such as GD, HT, PT, and PPT in some patients and activate otherwise dormant diseases into a clinically recognizable state in others (25–28).

Apoptosis of the thyrocytes was demonstrated in autopsy material from patients with SARS during the 2002 outbreak (36). In these specimens, SARS-CoV per se was not found in the thyrocytes and the follicular structure was mostly preserved, which suggested that direct viral invasion into the cell was not the cause of the apoptosis. Instead, autoimmune provocation or activation, triggered by the cytokine storm during SARS-CoV infection was likely to be causal for the thyroid damage. The pathological findings in the three cases of COVID-19 were essentially the same as those in patients with SARS-CoV, i.e., apoptosis of follicular cells in the absence of the virus itself in the thyrocytes (67), suggesting the operation of a similar, cytokine-mediated thyroid insult in SARS and COVID-19. Recently, Campi et al. found that the majority of patients with COVID-19 had normal thyroid function but that low TSH levels were seen either at admission or during hospitalization in 39% of patients, associated with low FT3 in half of the cases. They concluded that the thyroid dysfunction is likely due to the cytokine storm induced by SARS-Cov-2 with a direct or mediated impact on TSH secretion and deiodinase activity, and not likely a destructive thyroiditis (68).

Of note is that lymphopenia was observed in a case of SARS-CoV-2 before the development of PPT, which occurred 4 months after the delivery (28). By the time of the PPT, her lymphocyte count had returned to normal. Such an alteration in the number of lymphocytes in the general circulation was compatible with the idea of the causal link between the SARS-CoV-2 infection and the development of PPT (28).

#### Provocation of Thyroidal Autoimmunity and the Human Leukocyte Antigen System

Human Leukocyte Antigen (HLA) plays an important role in the immune system at the interface of antigen presentation by the T-cells and its recognition and antibody production by the B-cells (51) (**Figure 2**). The HLA class I molecules are abundantly expressed in various cells and induce CD8+T-cell response. On the other hand, the expression of HLA class II molecules is limited to antigen-presenting cells and responsible for CD4+ T-cell response. Because of polymorphism of HLA genes, divergent immune responses toward the same antigen and varied disease susceptibility are to be generated.

Increased prevalence of certain HLA genotypes in COVID-19 has been reported in certain populations including Chinese and Italian patients (69–71). The HLA gene and its polymorphisms have been implicated for many years (over 25 years) in AITD and SAT (72–74) and now in COVID-19 (75) (**Figure 2**). Higher incidence of SAT in those with the HLA-B35 or HLA-B67 antigens were reported (72). In 1974, increased frequency of the HL-A8 antigen in the GD patient population was reported (73). To date, Tomer and Davies comprehensively reviewed HLA class I and class II association with AITD in wide varieties of ethnics (74). Illustrating the possible association, a Japanese patient with PPT and COVID-19 (28) possessed the same HLA alleles predisposing to AITD (HLA-DP5 and HLA-B51) in the Japanese (51, 76).

### Minor Sequelae

#### Nonspecific Reaction to Severe Illness

Systemic critical diseases cause NTIS, in which the primary issue is a derangement in thyroid hormone metabolism. Alteration of the serum thyroid hormone concentration and its metabolites seen in COVIC-19 may well be added to the long list of causes for NTIS.

#### Angiotensin-Converting Enzyme 2 (ACE2) on Thyroid and Pituitary Cells

Angiotensin-converting enzyme 2 (ACE2) is the receptor utilized by SARS-CoV2 creeping into the cells, then initiating replication (77). In many organs including the lung, the gastrointestinal tract, the liver, the kidney, the skin, the heart, the hematopoietic cells, and the spleen, a direct cellular change by the virus was documented during COVID-19 (78). ACE2 expression has been known to impair thyroid function and also the function of the anterior pituitary gland during COVID-19 (77) So far, the existence of SARS-CoV-2 in the thyroid gland and pituitary gland is not clear by examinations of light microscopy, immunohistochemistry, electron microscopy, and quantitative RT-PCR (79). Furthermore, viral particles of SARS-CoV-2 were found in the frontal lobe of the brain and brain capillary endothelial cells (80). The SARS-CoV-2 infection has been known to impair olfaction and taste sense by affecting the central nervous system (81–83). Although clear evidence is lacking, infection of the thyrocyte, thyrotroph and corticotroph may result in lowered T3, T4, TSH, ACTH and cortisol. The dysregulation of the hypothalamic-pituitary-thyroid axis has been considered at least in part responsible for hypothyroidism in COVID-19 (17, 24, 27) (**Figure 2**).

## Future Perspectives and Conclusions

The case histories reported in the literature indicate that thyroidal disorders associated with COVID-19 such as SAT, PT, PPT are usually self-limiting, and GD and HT can be easily treated with thiamazole and L-T4, respectively. The beneficial effect of thyroid hormone supplements for patients who exhibit low FT4 and FT3 levels with NTIS and/or central hypothyroidism remains to be established.

Management would be more difficult if drug-induced agranulocytosis were to take place (but fortunately not so far reported), or thyroid storm (84), or myxedema coma were to occur (85). As shown here, our understanding of the thyroidal manifestation of COVID-19 is far from complete as is the etiologic view of COVID-19 and thyroid insults. Although case reports are definitely important in helping us understand the association, future research, hopefully in a prospective manner with longitudinal analyses, is required. This would involve the histologic and cytological examination of the thyroid gland in a large number of patients in order to identify direct evidence regarding the nature and cause of thyroid damage with the COVID-19 virus and the detailed immune response in those patients with thyroid dysfunction. Of particular interest is the need to clarify if thyroid autoimmunity in COVID-19 is an innocent-bystander or another culprit in its severity (86).

The strength of this review is its high originality and systematic description. For the first time, we systematically explained the detailed pathophysiology of the thyroid abnormalities associated with SARS-CoV2 infection, firmly based on the accumulated evidence in the literature. The limitation of this paper is that several review articles dealing with thyroid disorders related to COVID-19 have been already reported (30–32).

## Author Contributions

HI and TA contributed to conception, design, searching publications, writing the manuscript. All authors contributed to the article and approved the submitted version.

## Funding 

This work was partially supported by Takeda Science Foundation.

## Conflict of Interest

The authors declare that the research was conducted in the absence of any commercial or financial relationships that could be construed as a potential conflict of interest.

## Supplementary Material

Supplementary Figure 1 An overview on the postulated mechanism for the thyroid insults with COVID-19. Tier 1: The viral infection causes derangement in immune system, of which dysregulation of the T-cell system (including regulatory T-cell) and resultant cytokine overproduction and storm affect the thyroid gland. Tier 2: Cellular and humoral immunity in an around the thyroid is provoked or activated: the subjects having autoimmune thyroid disorders or those with a particular set of human-leukocyte antigen genotype may be vulnerable. Tier 3: Imbalanced provocation and/or activation of Th1, Th2 and other subtypes of T/B cells occur, leading to the thyroidal stimulation, destruction and suppression, or a combination thereof. The thick arrow in the right end implies a mutual stimulation between Th1 and Th2 systems. Tier 4: Patients may present with the symptoms such as fatigue and lethargy, and a goiter whichmay be painful and is accompaniedwith fever if the destruction is prominent. Laboratory examination is needed for the correct diagnosis. There is a general temporal tendency as to the timing between the viral infection and the clinical onset of the thyroid abnormality (See **Figure 1**). Th1, human type 1 helper cell; Th2, human type 2 helper cell; TRAb, anti-TSH receptor antibody; TgAb, anti-thyroglobulin antibody; TPOAb, antithyroperoxidase antibody; GD, Graves’ disease; SAT, subacute thyroiditis; PT, painless thyroiditis; PPT, post-partum thyroiditis; HT, Hashimoto’s thyroiditis. In this and the following Figures, only Major Sequels of COVID-19 are depicted (see Text).

## Abbreviations

COVID-19, coronavirus disease 2019; SARS-CoV-2, severe acute respiratory coronavirus 2; TSH, thyrotropin; T3, triiodothyronine; T4, thyroxine; SAT, subacute thyroiditis; GD, Graves’ disease; HT, Hashimoto’s thyroiditis; PT, painless thyroiditis; PTT, postpartum thyroiditis.
