# Supplementary figures and images for: Coronavirus Disease 2019 and the Thyroid - Progress and Perspectives
Source: Front Endocrinol (Lausanne). 2021 Jun 24;12:708333. doi: 10.3389/fendo.2021.708333 (PMC8279745; doi:10.3389/fendo.2021.708333)

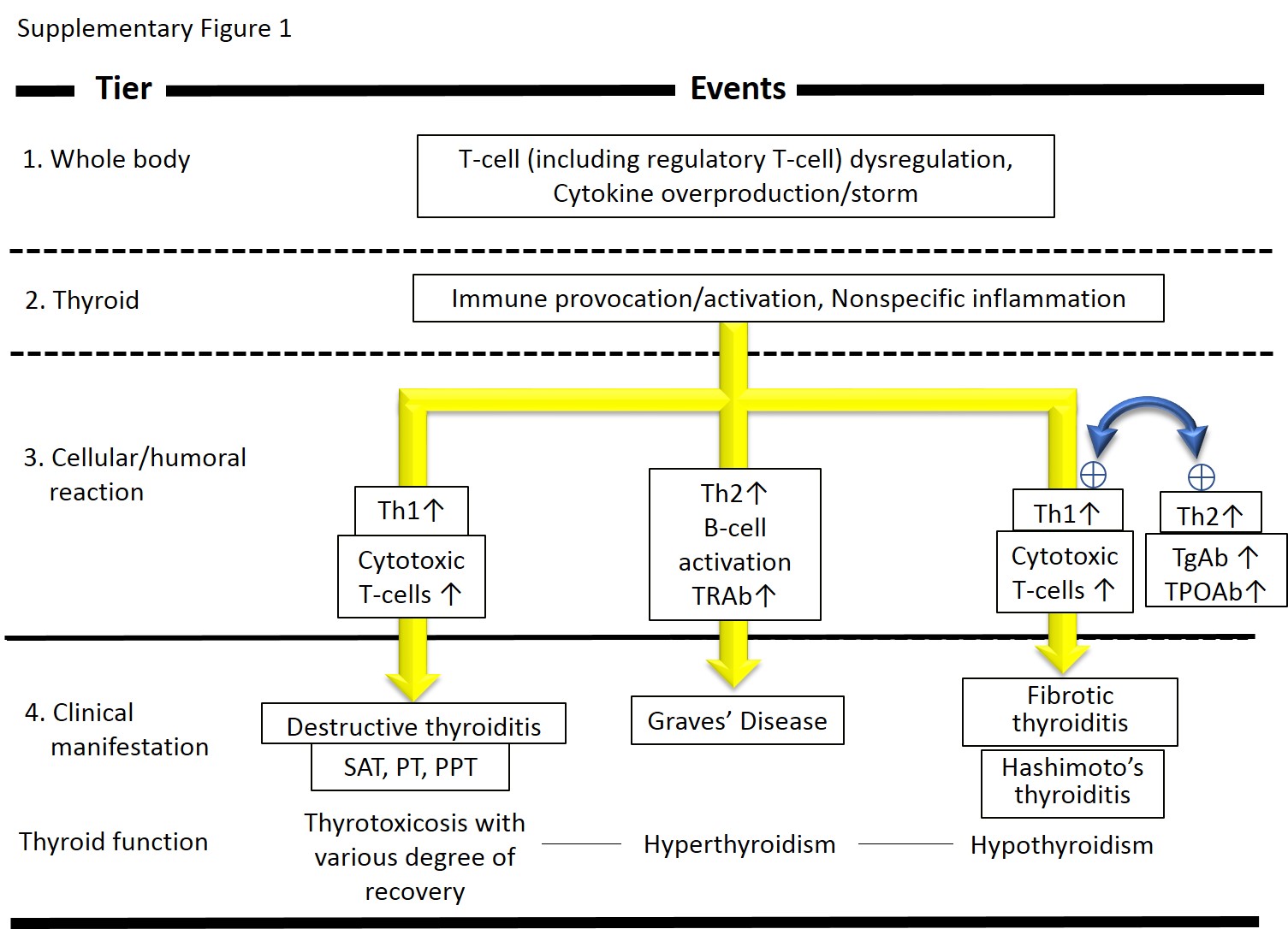

Supplement: Supplementary Figure 1 — An overview on the postulated mechanism for the thyroid insults with COVID-19. Tier 1: The viral infection causes derangement in immune system, of which dysregulation of the T-cell system (including regulatory T-cell) and resultant cytokine overproduction and storm affect the thyroid gland. Tier 2: Cellular and humoral immunity in an around the thyroid is provoked or activated: the subjects having autoimmune thyroid disorders or those with a particular set of human-leukocyte antigen genotype may be vulnerable. Tier 3: Imbalanced provocation and/or activation of Th1, Th2 and other subtypes of T/B cells occur, leading to the thyroidal stimulation, destruction and suppression, or a combination thereof. The thick arrow in the right end implies a mutual stimulation between Th1 and Th2 systems. Tier 4: Patients may present with the symptoms such as fatigue and lethargy, and a goiter whichmay be painful and is accompaniedwith fever if the destruction is prominent. Laboratory examination is needed for the correct diagnosis. There is a general temporal tendency as to the timing between the viral infection and the clinical onset of the thyroid abnormality (See Figure 1 ). Th1, human type 1 helper cell; Th2, human type 2 helper cell; TRAb, anti-TSH receptor antibody; TgAb, anti-thyroglobulin antibody; TPOAb, antithyroperoxidase antibody; GD, Graves’ disease; SAT, subacute thyroiditis; PT, painless thyroiditis; PPT, post-partum thyroiditis; HT, Hashimoto’s thyroiditis. In this and the following Figures, only Major Sequels of COVID-19 are depicted (see Text). [file Image_1.jpeg]
